# 

**Published:** 1823-06

**Authors:** 


### MONTHLY CATALOGUE OF MEDICAL BOOKS.

Medical Jurisprudence. By J. A. PARIS, M.D. F.R.S. F.L.S. Fellow of the Royal College of Physicians; and J. S. M. FONBLANQUE, Esq. Barrister at Law. In three volumes.—8vo. London, 1823.

A Treatise on Vaccination. For the Perusal of Parents. By J. C. YEATMAN, Surgeon Ext. to H. R. H. the Duke of Glocester, &c. &c.—8vo. Frome, 1822.

*We have received Notices of other Books being published, but, as the Works themselves were not sent, we cannot announce them.*

### NOTICES TO CORRESPONDENTS.

*Some of the Communications announced last month, and various others since received, are unavoidably postponed: they shall receive the earliest possible insertion.*

### INDEX

OF

THE FORTY-NINTH VOLUME

OF THE

### London Medical and Physical Journal.

PAGE

ABSORPTION, observations on . 13
Account of the Medical Schools of Berlin . . 171, 263
Account of a Structure, *sui generis*, in Birds . . . 5
——— of the New Faculty of Medicine, at Paris . 268, 357
Acid formed in distilling the Citric Acid . . . 356
Antidotes to various Poisons . 312
Aneurism of the Subclavian Artery 309
Apertures made in the Chest, Experiments on . . 479
Apoagnum Androsifolium, its Use in Medicine . . . 43
Artery, subclavian, tied . 46
———, external Iliac, ditto . ib.
Arteria Innominata, Ligature of . 475
Arsenic, poisoning with . 117, 439
Axilla, Tumours of the . 532

Belladonna, its use in Neuralgia . 42
Birds, structure, *sui generis*, in . 5
——— of Spine in . 4
Bills of Mortality . . 269
Biography of Dr. Marcet . 85
Biographical Account of Dr. Blair 88
——— of Dr. Currey 89
——— of Dr. Pett 176
——— of Dr. Jenner 271
——— of Dr. Haighton . . . . 446
——— of Mr. Charlton ib.
Black Urine, cases of . . 31
Bladder, Extraction of Stones from 533
Boulimia, case of . . 531
Books, Catalogues of 90, 177, 359, 448, 536
Bones of a Fœtus voided *per anum* 353
Botany, progress of . . 64
———, ditto Medical . . 268
Brain, Diseases of . . 6
———, wanting . . 5
Bronchocele, cured by tying the Arteries . . . 49

PAGE

Bronchotomy, cases of . 50, 51
Calcareous Concretions in the Choroid Plexus . . 43
——— in the Veins ib.
Calculi, method of breaking down 354
Calculus discharged by the Mouth 468
Camphor, use of, in Inflammations . 39
Cancer, use of Cold in . . 53
Carbonic Acid, Waters containing 533
Case of Boulimia . 531
Cases of Puerperal Convulsions . 266
Case of extra-uterine Gestation . 311
——— of perforation of the Perinium 61
Cases of Croup treated with cold Effusion . . . 31
——— of poisoning with Opium . 119
——— of Rupture of both Ventricles 9
——— of Alvine Concretions . 13
——— of Neuralgia, 109, 111, 281, 372, 471
——— of Paraplegia 187, of Petechiæ 286
Castor Oil, method of obtaining 356
Cataract, new Instrument for . 441
Catalogues of Books, 90, 177, 359, 448, 536
Cæsarean Section, cases . 55, 91
Cinnabar . . . 443
Chromate of Potass a Test for Arsenic 63
Chronic Ulcers, method of treating 354
Colica Pictonum, vinegar in the . 533
Combination of Acetic Acid with volatile Oils . . . 62
Condensation of the Gases . 443
Contributions to Morbid Anatomy 382
Croton Tiglium, on the preparation of . . . 398
Croup, cases of . 31, 304
——— cured by Sulphate of Copper 437
Curious Affection of the Heart . 436
Curvatures of the Spine . 96, 179

Detection of Poisons . . 268
Dislocations, Sir A. Cooper on 131, 219
Distention of the Intestines, case of 11
Distortions of the Spine, Mr. Bampfield on . . 96, 179

PAGE

Distortions of the Spine, Mr. Ward on 65
Double Uterus and Vagina . 529
Dropsy, Dr. Ernest's case of . 297
Duodenum, case of Imperforate . 468
Dysentery, treatment of . 438

Effluvia, effects of . . 17
Elaterium, medical powers of . 476
Emphysema, observations on . 30
Enlargement of the Stomach, case of . . . . 10
English Opium . . . 444
Epidemic Small-pox . . 33
Errata . . 90, 272, 360
Escape of the Liquor Amnii . 60
Ether Nitric, new method of preparing . . . 534
Extracts, Apparatus for making . 444
Experiments relative to Yellow Fever . . . 445
——— on the Cerebrum and Cerebellum . . 264
——— on Transudation . 352
——— on Absorption . 13
——— on the Nerves . 463
Extensive Injury of the Brain . 26
External Iliac, Ligature of the . 46
Extirpation of the Testicle . 58
Extra-uterine Conception, case of 311
Eye, new Muscle connected with the 2
——, Minute Structure of . ib.
——, motions of the . . 529

Faculty of Medicine of Paris 268, 357
Fever, observations on . 373
Fœtus affected with dry Gangrene 353
———, Bones of, voided *per anum* ib.
Foreign Body seven years in Trachea 436
Fractures, remarks on . 51, 52

Gall-bladder wanting . . 12
Gases, condensation of the . 443
General Dropsy, case of . 297
Generation, remarkable formation of the organs of . . 173
Gold, its use in medicine . 44

Half-yearly Report of the Small-pox Hospital . . . 168
Heart, curious affection of . 436
Heat, phenomena connected with 61
Hemorrhage, Uterine, observations on . . . . . 58
Hooping-Cough, use of Leeches to the forehead in . . 203
Hydriodate of Potass, method of preparing . . . 355
Hydrocyanic Acid, medical history of . . . . 125
Hypochondriasis, pathology of . 73

PAGE

Jaundiced Persons, Xanthöopia of 531
Iliac Artery, ligature of . 46
Inflammation of the Cerebellum . 25
Instances of Longevity . . 269
Iodine, observations on the use of 265
Iron, Prussiate of, its use . 43
Irritability of the Tongue . 530

Larynx, Rupture of the . . 29
Life of Fœtus, new means of ascertaining . . . 58
Ligature of Arteria Innominata . 475
Liquor Amnii, escape of . 60
——— Hydrar. oxymuriatis . 64
Lithotomy, cure of . . 116
———, new method of performing the operation of . 440
Longevity, instances of . 269

Mechanism of the Spine in Birds . 4
Medical Botany . . . 268
——— Schools at Berlin 171, 263
——— Report of the Neilgherry Mountains . . 211, 305
Medicine, new Faculty of, at Paris . . 268, 357
Meteorological Tables 89, 178, 270, 360, 447, 536
Method of breaking Urinary Calculi 354
——— of treating Chronic Ulcers ib.
Monthly Catalogue of Books, 90, 177, 359, 448, 536
Monument to Dr. Jenner . 359
Morbid Anatomy, contributions to 382
Motions of the Eye . . 529
Mouth, Calculus discharged by the 468
Muscles, unusual number of . 529

Neilgherry Mountains, Report on the . . . 211, 385
Nerves, observations on the Functions of the . . 18, 29
———, on the superadded . 449
———, experiments on the . 463
Neuralgia Cases, 109, 111, 281, 372, 471
New acid formed by distilling Citric Acid . . . . 356
—— kind of Truss . . 176
Nitric Ether, new method of preparing . . . 534
Notices to Correspondents, 90, 272, 360, 448, 536

Obituaries 85, 88, 89, 176, 271, 446
Œsophagus wanting . . 529
Oil, Castor, method of preparing 356
—— of Turpentine, its uses . 42
Operation of Lithotomy, new method of performing . 446

PAGE
Operation for Aneurism of Subclavian Artery . . 309
Opium (English) . . 444
Opthalmia, use of camphor in . . 39
Osteo-sarcoma of jaw . . 45
Osteology, system of . . 80
Oxalic Acid, Test for . . 266

Paralyses, partial . 23, 25
Paraplegia, fatal Case of . 387
———, cases of . . 187
Partial Paralysis of Retina . 532
Perineum, perforation of . 61
Perforation of Œsophagus . 434
——— the Stomach, cases of 12
Perspiration of the Feet, how restored . . . 354
Pin found in the Brain . . 26
Pneumonia, use of Tartar Emetic in 38
Pneumonia, remarks on . 368
Podophyllum Pellatum, its use . 43
Poisoning with Arsenic . 117, 439
——— with Opium . . 119
——— with Corrosive Sublimate 40
——— with Hydrocianic Acid . 41
———, means of emptying the Stomach in cases of . 40
Population of Russia . . 269
Prize Essay . . . 358
Progress of Medicine, &c. &c. from July 1822 to January 1823 1, 64
Prussic Acid, proper state of, for medical use . . 175
Prussiate of Iron, its use . 43
Puerperal Convulsions, cases of . 266
———, cured by Ergot . . . . 438
Purpura Hemorrhagica . 22, 286

Quarterly Reports of Diseases prevailing in London, 81, 166, 259, 350, 432, 527
Quinine, Sulphate of, its use . 43

Remarks on Abortion . . 217
——— on Uterine Hemorrhage 58
——— on Adhesion between the Brain and Dura-mater . 9
——— on Schirrus of the Brain 6
——— on Absorption . . 13
——— on Effluvia . . . 17
——— on Functions of the Nerves . . . 18
——— Transudation . . 352
——— Diseases of Tropical Climates . . . 289
Remarkable Formation of the Organs of Generation . . 173
——— Fact relative to Yellow Fever . . . 446
Reports of prevailing Diseases, 81, 166, 259, 432, 527

PAGE
Retina, partial Paralysis of . 532
——— Small-pox Hospital . 168
Rheumatism, translation of, to heart 215
Rupture of both Ventricles, case of 9
Russia, Population of . . 269

Small-pox Epidemics . 33, 36
Spine, Structure of, in Birds . 4
Stethoscope, application of, in pregnancy - - - 58
Stones, extraction of, from Bladder 533
Stramonium, use of, in Rheumatism 43
Structure of the Eye . . 2
Subclavian Artery, ligature of . 46
Sulphate of Copper in Croup . 437
——— of Quinine, its use . 43
Superfœtation, case of . . 442
Syphilitic, Ulcers of the Larynx, 273, 361
Syphilis, use of Gold in . 56

Tables, Meteorological, 89, 178, 270, 360, 447, 536
Tennessee, disease prevalent at, from eating the flesh of certain animals . . . 32
Testicle, extirpation of . . 58
Tetanic Affection, case of . 380
Tic Douloureux, cases of, 109, 111, 281
Tiglii Oleum, its uses . . 128
Tongue, Irritability of . . 530
Trachea, foreign body seven years in . . . . 436
Translation of Rheumatism to Heart 215
Transudation, observations on . 352
Treatise on Dislocations, by Sir A. Cooper . . 131, 219
Trepan, case of . . . 441
Truss, new kind of . . 176
Tumours of the Axilla . . 532

Ulcers of Larynx . . 273, 361
Urethra, instrument for dilating, in the female . . . 55
Uterine Hemorrhage, observations on . . . . 58
Uterus, extirpation of neck of . 54
——— and Vagina, double . 529
Vaccine Vesicle, curious case of 122
Vaccination in the Roman States 268
Vertebræ, part of, removed . 53
Vinegar in Colica Pictonum . 533
Volatile Oil of bitter Almonds . 175
Waters containing Carbonic Acid 533
Whiskers in feline and other Animals, use of . . 397
Xanthöopia of Jaundiced Persons 531
Yellow Fever, experiments relative to . . . . 445
———, remarkable fact relative to . . . . . 446

### REVIEWS.

### DOMESTIC.

PAGE
Practical Observations on Distortions of the Spine, Chest, and Limbs, &c. &c. by William Ward, F.L.S. . . 85
A Treatise on Dislocations, and on Fractures of the Joints, by Sir A. Cooper, Bart. &c. 131, 219
Historical Sketch of the Opinions entertained by Medical Men respecting the Varieties and secondary Occurrence of Small-pox, by John Thomson, M.D. F.R.S.E. &c. &c. . 233, 318
Practical Observations on the Treatment and Cure of Pulmonary Consumption, and on the Effects of the Vapour of boiling Tar in that Disease, by Sir A. Crichton, M.D. F.R.S. &c. . . . . 327, 336
History and Method of Cure of the various Species of Epilepsy, by John Cooke, M.D. . 408
Elements of the Theory and Practice of Physic, designed for Students, by George Gregory, M.D. . . . . 496
Essay on the Medicinal Efficacy and Employment of the Bath Waters, by Edward Barlow, M.D. . . . . 506

### FOREIGN.

PAGE
De l'Hypochondrie et du Suicide, par J. P. Falret, Docteur en Medicine, &c. 72, 150, 239
M. Scarpa and Mr. Briggs on Cancer and Schirrus . 337
Letters of Professor Vacca on the Ligature of the large Arteries 240
Rapport fait a l'Academie des Sciences, par M. Cuvier; sur un Memoire de M. Flourens, intitulé " Determination des Propriétes du Systeme Nerveux, ou Recherches Physiques sur l'Irritabilité et la Sensibilité . . . . . 517

### BIBLIOGRAPHY.

Dr. Paris's Pharmacologia . . . . . . . 78
Pharmacopœia . . . . . . . . . 79
A System of Osteology . . . . . . . . 80
Dr. Venable's Lecture on Oxalic Acid . . . . . 159
Dr. Price on Sangui-suction . . . . . . . 161
Dr. Herholdt's Case of Needles extracted from a Female . . 164

### MISCELLANEOUS.

### OBITUARY.

| | PAGE |
|---|---|
| Biography of Dr. MARCET | 85 |
| Biographical Notice of Mr. BLAIR | 88 |
| ——— Dr. CURREY | 89 |
| ——— Dr. PETT | 176 |
| ——— Dr. JENNER | 271 |
| ——— Dr. HAIGHTON | ~~446~~ 359 |
| ——— Mr. CHARLTON | ib. 446 |

| | |
|---|---|
| Account of the Medical Schools of Berlin | 171, 263 |
| New Faculty of Medicine of Paris | 268, 357 |
| Prize Essay | 358 |
| Provincial Monument to Dr. JENNER | 359 |
| Notice of the Society for Relief of Widows and Orphans | 446 |
| Annual Report of the National Vaccine Establishment | 535 |

METEOROLOGICAL TABLES.

Pages 89, 178, 270, 360, 447, 536.

MONTHLY CATALOGUE OF BOOKS.

Pages 90, 177, 359, 448, 536.

NOTICES TO CORRESPONDENTS.

Pages 90, 272, 360, 448, 536.

### REFERENCES TO PLATES.

| | |
|---|---|
| Cæsarean Operation | 90 |
| Distortion of the Spine | 178 |
| Calcareous Tumor connected with the Uterus | 384 |
| Mr. SHAW's Plates of the Nerves | 449 |
| Calculus discharged by the Mouth | 470 |

### NAMES OF AUTHORS,

*Of whose Works, Observations, &c. either a detailed Account, or more or less general View, is given in*

### THE FORTY-NINTH VOLUME OF THE LONDON MEDICAL AND PHYSICAL JOURNAL.

| | PAGE |
|---|---|
| AMESBURY, Mr. his observations on Fractures | 52 |
| AMUSAT, M. his instrument for breaking Calculi | 354 |
| ANDRAL, M. his cases of Schirrous Brain | 5 |
| ——— his observations on Dilatation of the Stomach | 10 |
| ARMSTRONG, Mr. his case of Rheumatism of the Heart | 215 |
| ASHBURNER, Dr. his case of Rupture of both Ventricles | 9 |
| AUMONT, M. his method of Extirpating the Testicle | 58 |
| AUTENRIETH, Dr. his observations on the Liver and Kidneys | 2 |
| AVISARD, M. his case of Excision of the Neck of the Uterus | 54 |
| BAKER, Dr. his case of Vaccine Vesicle occurring six months after the insertion of the Virus | 122 |
| BAMPFIELD, Mr. his Observations on the Spine | 96, 179 |
| BARON, Dr. his account of the Death of Dr. Jenner | 271 |
| BELL, Mr. C. his observations on the Functions of the Spinal Nerves | 18 |
| BELCOMBE, Dr. his case of Croup | 304 |
| BLAIR, Mr. obituary of | 88 |
| BÖDTCHER, Dr. his observations on the use of Camphor in Inflammation | 39 |
| BRESCHET, M. his observations on Children without Brains | 5 |
| BRANDE, Mr. on the action of Magnesia on Jalep | 175 |
| BRIGGS, Mr. on Cancer | 337 |
| BRODIE, Mr. his case of Subclavian Aneurism | 309 |
| BROUGHTON, Mr. on the Use of Whiskers, &c. | 397 |
| ——— Experiments on the Nerves | 463 |
| CHURCHILL, Mr. his observations on Gonorrhœa | 57 |
| CLINE, Mr. his case of Removal of a portion of the Vertebræ | 53 |
| COATES, Dr. his experiments upon Absorption | 15 |
| COLEMAN, Dr. his observations on a Disease prevailing at Ohio | 32 |
| COOKE, Dr. on Epilepsy | 408 |
| COOPER, Sir A. his Instrument for dilating the Female Urethra | 55 |
| ——— his Treatise on Dislocations | 131, 219 |
| ——— Dr. on Chromate of Potass as a Test of Arsenic | 63 |
| COPLAND, Dr. his case of Black Urine | 31 |
| CRAIGIE, Dr. his observations on the Pathology of the Brain | 9 |
| CRICHTON, Sir A. his observations on the Use of Tar Vapour in Phthisis | 36, 327 |
| CRAUFORD, Dr. his observations on Carbonate of Iron in Neuralgia | 109 |
| CURRY, Dr. obituary of | 89 |
| DAVY, Dr. on the Liquor Hydrargyri Oxymuriate | 64 |
| DOBEREINER's Apparatus for making Extracts | 444 |
| ———, M. on the Formation of Formic Acid | 62 |
| DOUBLE, M. on Sulphate of Quinine | 43 |
| DOUGLASS, Dr. his case of Rupture of the Perinæum | 60 |
| DUBLANE, M. his observations on the Action of Mercury | 17 |
| DUCACHET, Dr. his case of Re-union produced by a Seton | 53 |
| DUNCAN, Dr. jun. his observations on Purpura | 22 |
| DUNGLISON, Mr. his observations on Inflammation of the Cerebellum | 26 |
| DUPONCHET, M. his case of Injury of the Brain | ib. |
| DUPUY, M. his case of Croup in a Dog | 31 |

### *Names of Authors.*

PAGE

EARLE, Mr. H. his observations on the Spine of Birds . . . 4
EDWARDS, Mr. his case of Poisoning by Arsenic . . . 117
ERNEST, Dr. his Case of Dropsy . . . . . . . 297
EVANS, Mr. on Croton Tiglium . . . . . . . 398
EYSENHARDT, M. his Observations on the Liver and Kidneys . . 2
FAGUOR, M. on the Formation of Castor Oil . . . . 355
FALRET, Dr. on Suicide and Hypochondriasis . . 72, 150, 239
FLOURENS, M. his experiments on the Cerebellum . . . 264
FOHMAN, Dr. his observations on the Absorbents . . . 16
FODERE', M. on Absorption, &c. . . . . . . . 352
FORBES, Dr. J. his observations on a Small-pox Epidemy at Chichester 33
——————— on Pneumonia . . . . . . . 368
GARTNER, Dr. his account of a Duct between the Uterus and Ovaries in various Animals . . . . . . . . . 5
GOOCH, Dr. observations on Uterine Hæmorrhage . . . 59
GREEN, Mr. his case of Cæsarean section . . . . . 55
GREGORY, Dr. G. his case of Cynanche Cellularis . . . 28
——————— Statistical Reports . . . . . 166, 168
GRAAFE on Ligature of the Arteria Innominata . . . . 475
GROENING, M. his application of the Thermometer to Distillation . 63
HARRISON, Dr. observations on the Spine . . . . 187
HAVILAND, Dr. his case of Ulceration of the Stomach . . . 12
HARDEN, Dr. his observations on Cold Affusion in Croup . . . 39
HILL, Dr. his observations on Syphilis . . . . . 57
HOME, Sir E. his observations on the Eye . . . . . 2
HORNER, Dr. his description of a new Muscle . . . . ib.
HORN, MM. their case of Cæsarean Section . . . . 55
HAWKINS, Mr. on Ulcers of the Larynx . . . . 273, 361
HUBBARD, Dr. his experiments on Absorption . . . . 14
HUFELAND, his observations on Tar Vapour . . . . 37
HUTCHINSON, Mr. on Oil of Turpentine in Tetanus . . . 114
——————— on Carbonate of Iron in Neuralgia . . 281, 471
HARRISON, Dr. his fatal case of Paraplegia . . . . 387
JAMES, Dr. J. his observations on Small-pox . . . 35
JEFFREYS, Mr. his cases of Neuralgia . . . . . 199
JOHNSON, Dr. James, case of enlarged Kidney . . . . 12
——————— Charles, on the application of a Ligature to the inverted Uterus 54
JUKES, Mr. his method of evacuating the Stomach . . . 40
JOHNSON, Mr. on Fever . . . . . . . . 373
JEFFREYS, Mr. his case of Neuralgia . . . . . 372
——————— case of imperforate Duodenum . . . . 468
KERGARADEE, M. his application of the Stethoscope to ascertain the life of the Fœtus in utero . . . . . . .
KINGLAKE on the Medical Powers of Elaterium . . . 476
LAWRANCE, Dr. his experiments on Absorption . . . . 15
MACLEOD, Dr. his Statistical Reports . . . . 259, 527
——————— his case of Black Urine . . . . . . 31
M'CALL, Dr. his observations on a disease prevailing at Tennessee . 32
MAJENDIE, his observations on the functions of the Spinal Nerves . 18
MAPPES, Dr. his observations on the Liver and Kidneys . . . 2
MARCET, Dr. obituary of . . . . . . . . 85
MAUNAURY, M. his observations on Rupture of the Membranes . 60
MONEY, Mr. his observations on Oil of Turpentine . . . 42
——————— case of Lithotomy . . . . . . 116
MURRAY, Mr. on the Antidotes to Prussic Acid and Opium . . 41
MOTT, Dr. his case of Extirpation of the Jaw . . . . 45
NACQUART, M. case of Gall-bladder wanting . . . . 12
NICHOLL, Dr. W. his observations on Purpura . . . . 23
NIEL, Dr. his observations on Muriate of Gold . . . . 44
O'BRIEN, Dr. his case of ruptured Larynx . . . . . 29
ORTON, Mr. on the Topography of the Nielgherry Mountains . . 211
PARIS, Dr. his Pharmacologia . . . . . 78, 125, 128, 312

PAGE

**Paris**, Dr. his Medical Jurisprudence . . . . . . 487
**Percy**, M. Case of Superfœtation . . . . . . 442
**Pesseria**, M. on Prussic Acid . . . . . . . . 355
**Peschier**, M. his observations on Tartar Emetic in Pneumonia . . 38
**Philip**, Mr. R. on the Pulvis Antimonialis . . . . . 64
**Pinel**, M. jun. his observations on Induration of the Brain . . 7
**Pouillet**, M. his observations on Caloric . . . . . 61
**Pretty**, Mr. his case of Petechiæ . . . . . . . . 286
**Price**, Dr. on Sangui-suction . . . . . . . . 161
**Rousseau**, Dr. his observations on Odours and Effluvia . . . 17
**Rowley**, Mr. on Stricture, &c. . . . . . . . . 301
**Scarpa**, M. on Cancer . . . . . . . . . . 337
**Segalan**, M. his experiments on Absorption . . . . . 15
**Serres**, M. his observations on the Cerebellum . . . . . 25
**Shaw**, Mr. his observations on partial Paralysis . . . . . 23
——— his observations on Emphysema . . . . . 30
——— on the Nervous System . . . . . . . . 449
**Shonberg**, Dr. A. Von, his case of Malformation of the Genital Organs 173
**Swan**, Mr. his observations on the action of Mercury . . . 17
**Thomson**, Dr. on Small-pox . . . . . . . . 233
**Thompson**, Mr. on Belladonna in Neuralgia . . . . . 42
——— Mr. A. T. on Iron in Do. . . . . . . . 111
**Todd**, Mr. his observations on Diseases of the Lachrymal Gland . . 27
——— his cure of Subclavian Aneurism . . . . . 46
**Travers**, Mr. his account of the case of Dr. Pett . . . 176
**Toms**, Mr. case of Tetanic Affection . . . . . . 380
**Vacca**, Professor, on the Ligature of large Arteries . . . 420
**Vogel**, M. on Oil of Bitter Almonds . . . . . . 175
**Vaus**, M. his observations on the Heart . . . . . . 3
**Venables**, Dr. on Oxalic Acid . . . . . . . . 159
**Valentin**, M. case of Gall-bladder having two Ducts . . . 13
——— case of Pin found in the Brain . . . . . . 26
**Van-Buren**, Mr. his cases of Cæsarean Section . . . . . 91
**Vauquelin**, M. on the combination of Acetic Acid and Alcohol with Volatile Oils . . . . . . . . . . 62
**Waller**, Dr. on the use of Soda Tartarizata . . . . . 123
**Ward**, Mr. on the Spine . . . . . . . . . 65
——— Mr. H. W. on Absorption . . . . . . . . 217
**Webster**, Dr. his Statistical Reports . . . . . 81, 350
——— his observations on Hooping-cough . . . . . 203
**Wishart**, Mr. his case of Tumors of the Dura Mater, Skull, &c. . 8
**Wade**, Mr. contributions to Morbid Anatomy . . . . . 382
**Williams**, Dr. on Apertures made into the Chest . . . . 479
**Yeatman**, Mr. his cases of Poisoning by Opium . . . . . 119
**Zollickoffer**, Dr. his observations on various Medicines . . 43

ERRATA.

Pages 90, 272, 360, 448.

